# Correction: Constitutive scaffolding of multiple Wnt enhanceosome components by Legless/BCL9

**DOI:** 10.7554/eLife.27150

**Published:** 2017-03-30

**Authors:** Laurens M van Tienen, Juliusz Mieszczanek, Marc Fiedler, Trevor J Rutherford, Mariann Bienz

van Tienen LM, Mieszczanek J, Fiedler M, Rutherford TJ, Bienz M. 2017. Constitutive scaffolding of multiple Wnt enhanceosome components by Legless/BCL9. *eLife* **6**:e20882. doi: 10.7554/eLife.20882.

Published 15, March 2017

On publication of our article, we noticed that a penultimate version of Figure 4 was inadvertently resubmitted instead of the final version, along with the penultimate version of the accompanying Supplementary file 2. The final version of Figure 4 is subtly different and is shown below.

In addition, the sentence in the Methods describing SuperTOP assays should be slightly modified, as follows:

‘For luciferase reporter assays, SuperTOP (Veeman et al., 2003) was co-transfected with CMV-Renilla as internal control, and assays were performed initially 48 hours post-transfection, but subsequently 24 hours post-transfection (for all figures shown in this study)’.

The original sentence is given below, for reference:

‘For luciferase reporter assays, SuperTOP (Veeman et al., 2003) was co-transfected with CMV-Renilla as internal control, and assays were performed 48 hours post-transfection’.

These corrections do not affect in any way the conclusions of our study.

The corrected Figure 4 is shown here:

**Figure 1:**
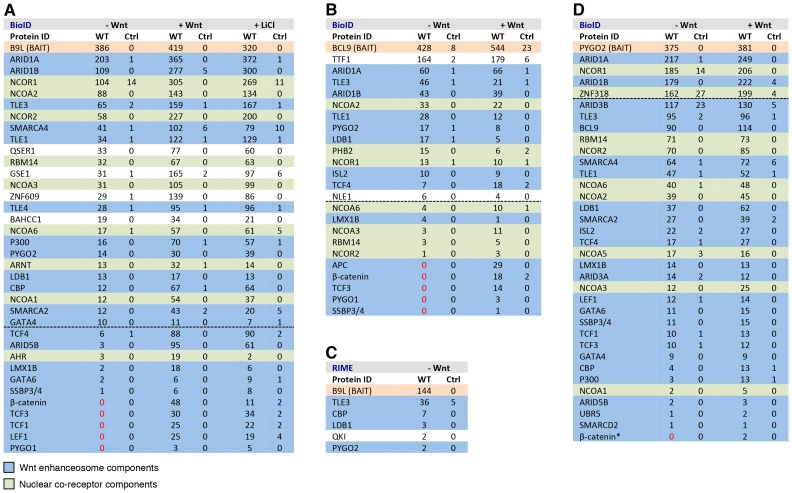

The originally published Figure 4 is also shown for reference:

**Figure 2:**
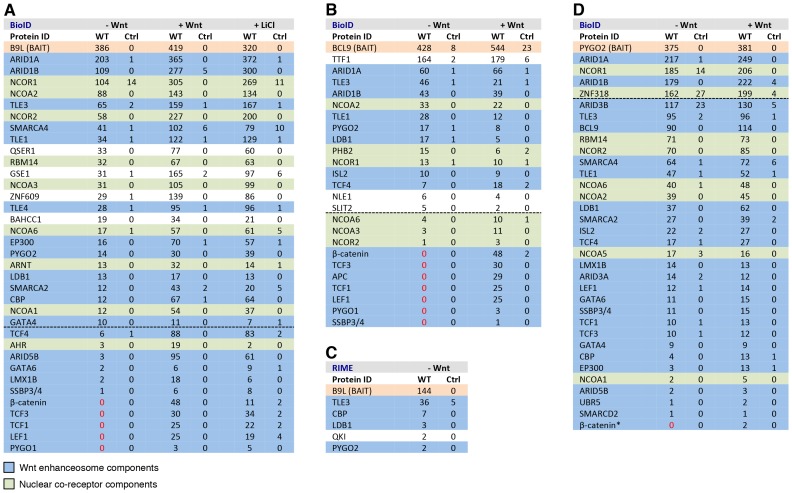

The article has been corrected accordingly.

